# Serum Angiogenesis Markers and Their Correlation with Ultrasound-Detected Synovitis in Juvenile Idiopathic Arthritis

**DOI:** 10.1155/2015/741457

**Published:** 2015-05-04

**Authors:** Joanna Świdrowska, Piotr Smolewski, Jerzy Stańczyk, Elżbieta Smolewska

**Affiliations:** ^1^Department of Pediatric Cardiology and Rheumatology, Medical University of Lodz, 91-738 Lodz, Poland; ^2^Department of Experimental Hematology, Medical University of Lodz, 93-513 Lodz, Poland

## Abstract

Synovial angiogenesis is considered to be an important early step in the pathogenesis of juvenile idiopathic arthritis (JIA). In this study we assessed levels of angiogenic markers in serum or synovial fluid and their possible relevance to disease activity or degree of ultrasound signs of synovial inflammation and angiogenesis in early JIA. The concentration of vascular endothelial growth factor (VEGF), its soluble receptors 1 and 2 (sVEGF-R1, sVEGF-R2), and angiopoietins 1 and 2 (ANG-1, ANG-2) were evaluated in 43 JIA patients and 23 healthy controls. Synovial angiogenesis was assessed by means of Power-Doppler Ultrasonography (PDUS), according to the fourth-grade vascularity scale. VEGF and its receptors' (sVEGF-R1, sVEGF-R2) serum levels were significantly higher in JIA patients (*p* = 0.002). We found large variation in serum ANG-1 and ANG-2 levels. The PDUS imaging identified increased synovial microvascular blood flow in 15 (35.7%) examined JIA children. Intensity of joint vascularization correlated with higher serum VEGF and its levels was lowest in grade 0 and highest in grade 3 (*p* < 0.007 and *p* < 0.001, resp.). In conclusion, the high correlation between synovial microvascular blood flow, serum angiogenic proteins, and symptoms of synovitis may indicate its important role in pathogenesis of JIA.

## 1. Introduction

Juvenile idiopathic arthritis (JIA) is the most common childhood chronic rheumatic disease, characterized by arthritis of unknown origin with onset before the age of 16 years [1, 2]. The inflammatory process in JIA is characterized by excessive proliferation of synoviocytes. It is strongly associated with a neovascularization due to increased metabolic requirement of hypertrophic synovium [3]. Neovascularization, also defined as angiogenesis, is a complex process in which new capillaries develop from preexisting vasculature due to hypoxemia, injury, or inflammation of the tissues. This process plays a pivotal role in a number of physiological and pathological conditions, including chronic inflammatory diseases [4]. Angiogenesis is regulated by the imbalance between proangiogenic and antiangiogenic factors [5] and occurs in the first stage of synovitis. Vascular endothelial growth factor (VEGF) is the best known and the most endothelial cell-specific angiogenic factor [6]. The VEGF family currently consists of six members: VEGF-A, VEGF-B, VEGF-C, VEGF-D, VEGF-E, and placenta growth factor. Soluble VEGF receptor-1 (sVEGF-R1; sFlt-1) regulates the biologic activity of VEGF and it is one of the three specific receptors that mediate the actions of VEGF [7]. Angiopoietins (ANGs), less explored in the pathogenesis of chronic inflammatory connective tissue diseases, are known as the mediators of angiogenesis regulating endothelial integrity and inflammation.

In adult rheumatoid arthritis (RA) VEGF has been described as a crucial factor of neovascularisation at the stage of hypertrophic synovium [8]. It has been found in higher concentrations in serum and synovial fluid and there was a correlation found between its levels and disease activity [9]. Now, there have been very few studies published investigating the production of VEGF in the inflamed joints of children with JIA.

Recently, visualization of hypertrophic synovium and its vasculature has become possible by high resolution ultrasound (US) imaging and the Power-Doppler (PD) mode (PDUS). Technical improvements of ultrasonography allowed detecting the low-velocity blood flow in synovial microvessels. High sensitivity of synovitis US in RA has been already shown in several publications considering adult rheumatology [10, 11]. Unfortunately, there are only few reports discussing that issue in pediatric rheumatology [12].

The purpose of the study was to discuss the role of serum vascular angiogenic factors: VEGF, its soluble receptors, and ANGs 1 and 2 (ANG-1, ANG-2, resp.) in pathophysiology of JIA. Importantly, we also aimed to investigate the intra-articular synovial microvascular blood flow in clinically symptomatic JIA joints by means of PDUS in correlation with biological markers of inflammation and angiogenic factors.

## 2. Materials and Methods

### 2.1. Patients

Forty-three children who met the International League of Associations for Rheumatology (ILAR) criteria for JIA [13], 31 girls and 12 boys, median age 9 years (range 1.5–17 years), were included into the study. All had an early diagnosis of JIA determined by a consultant pediatric rheumatologist. All children were without any oral/iv corticosteroid history. None of children had received any intra-articular steroid injection in the past. The control group consisted of 23 healthy children, 15 girls and 8 boys, median age 10.5 (range 4–17.5). The study was approved by the local Ethics Committee.

### 2.2. Clinical and Laboratory Assessment

Each child had a detailed clinical history taken. The following data were recorded for each patient at the study visit: sex, age, ILAR category, disease duration, current treatment, and active joints count. A joint with active disease was defined as the presence of swelling or tenderness/pain on motion and restricted motion [14]. Activity of JIA was established based on the 27-joint Juvenile Arthritis Disease Activity Score (JADAS-27) [15]. Low, intermediate, and high stages of the disease activity have been distinguished accordingly. In the study group 27 (62.8%) children had low, 10 (23.25%) medium, and 6 (13.95%) high activity of the disease (Table 1). All the examined patients had a normal renal function. Majority of the study group comprised children with oligoarthritis type of JIA onset (30 patients, 69.8%), whereas 13 children (30.2%) represented polyarticular and only 3 (6%) systemic disease. The type of onset was defined according to ILAR criteria (2001) [16].

Clinical evaluation was performed by two pediatric rheumatologists prior to laboratory testing or ultrasound examination. Laboratory assessment included determination of erythrocyte sedimentation ratio (ESR) by Westergren method and C-reactive protein (CRP) level using the immunoturbidimetric method. Normal values for these laboratory methods were for ESR <12 mm/h and for CRP 0–5.0 mg/dL. Results of antinuclear antibodies (ANA) and rheumatoid factor (RF) were also recorded, using standard methods.

### 2.3. VEGF, sVEGF-R1, sVEGF-R2, ANG-1, and ANG-2 Levels

Levels of human VEGF, human angiopoietins (ANG-1, ANG-2), and human soluble VEGF receptors 1 and 2 (sVEGF-R1 and sVEGF-R2, resp.) were measured by ELISA in serum samples from JIA patients and 23 age-matched healthy controls. Moreover, in 8 patients concentrations of these proteins in synovial fluid were measured. VEGF, sVEGF-R1, sVEGF-R2, ANG-1, and ANG-2 were assessed using a standard quantitative sandwich ELISA (Quantikine, R&D Systems, Lille, France) according to manufacturer's instructions.

### 2.4. Ultrasonography (US)

**Figure 1:**
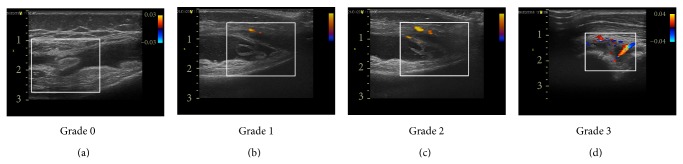
Ultrasound evaluation of joint vascularization scale using Power-Doppler Ultrasonography imaging. Examples of visualization of different degrees of joints inflammation and angiogenesis. Joint vascularization scale: grade 0 = no signs of vascularization, 1 = mild (presence of single/vessel dots), 2 = moderate (presence of confluent vessel dots in less than half of the synovial area), and 3 = marked (presence of confluent vessel dots in more than half of the synovial area).

US assessment was performed in the same hospitalization with clinical and laboratory examination, by a clinician experienced in musculoskeletal ultrasonography. A Philips CX50 CompactXtreme ultrasound system and a 5–12 MHz linear transducer were used in this study. The examination was performed in a minimum of two planes (longitudinal and transverse) and included the following joints: knees, ankles, wrists, elbows, metacarpophalangeal joints (MCP), proximal interphalangeal joints (PIP), metatarsophalangeal joints (MTP), and midfoot. The diagnosis of US-detected synovitis using gray-scale US was defined by the presence of synovial hypertrophy and/or the presence of a compressible anechoic space within the joint, representing fluid [17]. Synovitis detected by US was graded as mild, moderate, or severe (score from 0 to 3). Hypertrophic synovium was scanned for the presence of Power-Doppler signal, standardized with a lower pulse repetition frequency of  750 MHz and low wall filters. The color gain was increased to the highest values not generating PD signals under the bony cortex [18]. PDUS signal was scored on semiquantitative four-grade scale: 0 = no signs of vascularization, 1 = mild (presence of single/vessel dots), 2 = moderate (presence of confluent vessel dots in less than half of the synovial area), and 3 = marked (presence of confluent vessel dots in more than half of the synovial area) (Figure 1).

### 2.5. Statistics

For the statistical analysis of data obtained, the range of the measured variable, mean, median, and standard deviation (SD) were calculated, using statistical software (STATISTICA v.7.0, Tulsa, OK, USA). The data are presented as mean ± SD values. The differences between values were evaluated with nonparametric Mann-Whitney test, where the distribution of data was normal. The correlation between features was evaluated using the Spearman rank coefficient *ρ*. The *p* values less than 0.05 were considered statistically significant.

## 3. Results

The characteristics of the examined group are shown in Table 1.

VEGF serum levels were significantly higher in JIA patients than in healthy controls (*p* = 0.002, Table 2). Similar pattern was observed for sVEGF-R1 and sVEGF-R2 (*p* = 0.004 and *p* = 0.001, resp.). There was large variation in serum ANG-1 and -2 levels in JIA patients and healthy controls, without statistically significant differences (Table 2).

In JIA children serum VEGF levels correlated significantly with CRP and ESR (*R* = 0.39 and *R* = 0.41, resp.; *p* < 0.05). Serum levels of ANG-1 correlated with white blood cells (WBC) and platelets (PLT) counts (*R* = 0.31 and *R* = 0.55, resp.; *p* < 0.05), whereas ANG-2 correlated with WBC count (*R* = 0.32 and *p* < 0.05). There was no correlation of neither sVEGF-R1 nor sVEGF-R2 levels and examined laboratory parameters (Table 3). There were no statistically significant differences in examined markers of angiogenesis and disease activity, type of JIA onset, and the disease duration.

Comparison between concentrations of angiogenic proteins in serum and synovial fluid showed significantly higher levels of VEGF and sVEGF-R1 in synovial fluid (*p* = 0.004 and *p* < 0.001, resp.), whereas sVEGF-R2 and ANG-1 were higher in serum of JIA children (*p* = 0.008 and *p* < 0.001, resp.). ANG-2 concentration in serum and synovial fluid was similar (Table 4).

**Figure 2:**
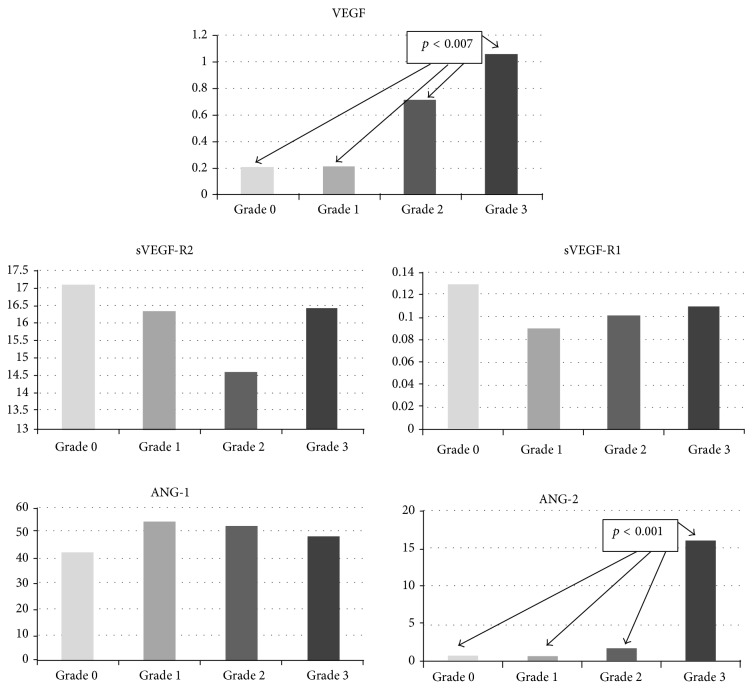
Ultrasound joint vascularization scale and serum levels of angiogenic proteins in JIA children. Joint vascularization scale: grade 0 = no signs of vascularization, 1 = mild (presence of single/vessel dots), 2 = moderate (presence of confluent vessel dots in less than half of the synovial area), and 3 = marked (presence of confluent vessel dots in more than half of the synovial area).

The PD mode identified marked synovial microvascular blood flow in joints (grade 3 vascularization) in 15 JIA patients (35.7%). There were no significant differences in angiogenic protein levels between grades 0 and 1 and grades 2 and 3. VEGF levels were lowest in grades 0/1 and highest in grade 3 (*p* < 0.007; Figure 2). ANG-2 levels were significantly higher in grade 3 than in 0, 1, or 2 (*p* < 0.001). Interestingly, sVEGF-R1 levels in grade 2 were statistically lower than in grade 0 (*p* = 0.030).

There were no statistically significant differences between angiogenic protein levels and radiological picture of joints in JIA children.

## 4. Discussion

Angiogenesis starts very early in rheumatoid process and is controlled by a variety of stimulators and inhibitors [19]. It is crucial to chronic inflammation and implies being essential pathogenetic mechanism in both the establishment and persistence of RA and, probably, JIA [20]. In this study we assessed five angiogenesis markers in patients with JIA. Among them were ANG-1 and ANG-2, to the best of our knowledge for the first time evaluated in JIA. Levels of these markers were correlated with signs of synovial inflammation measured using PDUS imaging.

Elevated serum levels of VEGF and its receptors have been previously shown in adult patients with RA [21, 22]. Among the angiogenesis stimulators, we found significantly higher serum VEGF levels and its soluble receptors (sVEGF-R1 and sVEGF-R2) in JIA children than in healthy controls. These results are in agreement with two other studies investigating VEGF in this disease [12, 23]. Moreover, Lee et al. [24] showed no correlation between VEGF levels in serum and synovial fluid from the same RA patient. Our study revealed the higher concentration of VEGF and sVEGF-R1 and, inversely, lower concentration of sVEGF-R2 and ANG-1 synovial fluid of JIA patients.

The other angiogenesis stimulators investigated in our study were ANG-1 and ANG-2. The role of ANG-1 is the stabilization of new capillaries, while the role of ANG-2 is antagonistic. Namely, ANG-2 blocks the vessel maturation [25]. As was mentioned, there are no studies considering the role of ANGs in JIA. Available data regarding ANGs' expression in adult patients with RA are unclear [26, 27]. We did not find significant differences in ANGs' serum levels between JIA and healthy children.

Besides the increase of some angiogenesis stimulators serum levels in JIA patients, our study showed also their correlation with biomarkers of inflammation. Interestingly, VEGF level was strongly correlated with CRP and ESR levels. In contrast, ANGs' levels corresponded with WBC and PLT count. Lee et al. [24] showed the similar pattern of VEGF and interaction of inflammation markers in RA patients.

Since US imaging and PDUS have clearly exposed the inflammation and blood flow in synovium, we analyzed whether there was a correlation between angiogenic markers and the US and PDUS findings. The PDUS identified marked synovial microvascular blood flow in joints (grade 3 vascularization) in more than one-third of JIA patients. Our study has also showed the significant relationship between VEGF levels and synovial vasculature blood flow. The lowest serum levels of VEGF were found in vascularity grades 0/1 and highest in grade 3. ANG-2 serum levels were also significantly higher in grade 3. Strunk et al. [28] and Gok et al. [29] in their studies on RA patients did not find any significant correlation between serum VEGF levels and the degree of synovial vascularity. In the same study, Gok et al. [29] found the positive correlation between ANG-1 levels with effusion, but not synovial and PDUS scores. To our knowledge, there are no other studies considering the correlation between PDUS vascularity grade scale and other angiogenic factors, such as VEGF receptors and ANG-2.

In conclusion, our study strongly suggests the crucial role of angiogenesis in JIA. Serum angiogenesis modulators are associated not only with pathogenesis of JIA, but also with its severity and progression. VEGF, as the most specific angiogenesis marker, reflects the degree of inflammation in JIA patients. In compilation with VEGF level, PDUS provides early information about the synovitis activity during the course of the inflammatory process. Although the lack of specificity makes the angiogenic modulators still not applicable for diagnostic principles, their close correlation with disease severity might be essential in JIA patients monitoring and follow-up.

## Figures and Tables

**Table 1:** Characteristics of the examined group.

JIA children	*N* (%)/Me (range)
Total number	43
Sex	
Girls	31 (72.1%)
Boys	12 (27.9%)
Age	9 (1.5–17) years
Time of JIA duration	7 (0.5–120) months
Type of onset	
Oligoarthritis	30 (69.8%)
Polyarthritis	13 (30.2%)
Disease activity	
Low	27 (62.8%)
Moderate	10 (23.25%)
High	6 (13.95%)
WBC	7.7 (3.0–19.0) G/L
PLT	355.0 (156.0–613.0) G/L
ESR	21 (2–130) after 1 h
CRP	2.58 (0.05–148.6)
ANA > 1 : 160	19 (44.2%)
RF > 14 IU	28 (65.1%)
US of joints^∗^	Grades 0–6 (13.95%), grades 1–8 (18.6%), grades 2–14 (32.55%), grades 3–15 (34.9%)
RTG of hands	Types 1–26, types 2–11, types 3–6

^∗^Joint vascularization scale: grade 0 = no signs of vascularization, 1 = mild (presence of single/vessel dots), 2 = moderate (presence of confluent vessel dots in less than half of the synovial area), and 3 = marked (presence of confluent vessel dots in more than half of the synovial area).

**Table 2:** Serum levels of angiogenic proteins in JIA in comparison to healthy children.

Parameter	Ctrl	JIA	*p*
	*N* = 23	*N* = 43	
VEGF	0.21 ± 0.16	0.64 ± 0.63	**0.002**
R1	0.09 ± 0.03	0.11 ± 0.03	**0.004**
R2	10.34 ± 4.47	15.92 ± 7.10	**0.001**
ANG-1	48.85 ± 16.39	50.01 ± 17.10	NS
ANG-2	1.81 ± 1.44	6.34 ± 34.12	NS

**Table 3:** Correlation between angiogenic proteins and laboratory parameters in JIA children.

Variable	VEGF	R1	R2	ANG-1	ANG-2
WBC	0.13	0.21	0.16	**0.31**	**0.32**
PLT	0.22	0.18	0.28	**0.55**	0.10
CRP	**0.39**	−0.07	−0.06	0.26	0.26
ESR	**0.41**	0.07	0.07	0.17	0.13

**Table 4:** Comparison between angiogenic protein levels in peripheral serum and synovial fluid.

Parameter	Serum	Synovial fluid	*p*
	*N* = 43	*N* = 8	
VEGF	0.64 ± 0.63	1.84 ± 1.54	**0.004**
sVEGF-R1	0.11 ± 0.03	0.46 ± 0.55	**<0.001**
sVEGF-R2	15.92 ± 7.10	8.70 ± 4.82	**0.008**
ANG-1	50.01 ± 17.10	5.78 ± 3.68	**<0.001**
ANG-2	6.34 ± 34.12	6.06 ± 7.81	NS
